# Prediction of Hypertension Based on Facial Complexion

**DOI:** 10.3390/diagnostics11030540

**Published:** 2021-03-17

**Authors:** Lin Ang, Bum Ju Lee, Honggie Kim, Mi Hong Yim

**Affiliations:** 1 Clinical Medicine Division, Korea Institute of Oriental Medicine (KIOM), 1672, Yuseong-daero, Yuseong-gu, Daejeon 34054, Korea; anglin2808@kiom.re.kr; 2 Korean Convergence Medicine, University of Science and Technology, 217, Gajeong-ro, Yuseong-gu, Daejeon 34113, Korea; 3 Future Medicine Division, Korea Institute of Oriental Medicine (KIOM), 1672, Yuseong-daero, Yuseong-gu, Daejeon 34054, Korea; bjlee@kiom.re.kr; 4 Department of Information and Statistics, Chungnam National University, 99 Daehak-ro, Yuseong-gu, Daejeon 34134, Korea; honggiekim@cnu.ac.kr

**Keywords:** CIELAB, facial variables, prediction models, chronic disease

## Abstract

This study aims to investigate the association between hypertension and facial complexion and determine whether facial complexion is a predictor for hypertension. Using the Commission internationale de l’éclairage L*a*b* (CIELAB) color space, the facial complexion variables of 1099 subjects were extracted in three regions (forehead, cheek, and nose) and the total face. Logistic regression was performed to analyze the association between hypertension and individual color variables. Four variable selection methods were also used to assess the association between hypertension and combined complexion variables and to compare the predictive powers of the models. The a* (green-red) complexion variables were identified as strong predictors in all facial regions in the crude analysis for both genders. However, this association in men disappeared, and L* (lightness) variables in women became the strongest predictors after adjusting for age and body mass index. Among the four prediction models based on combined complexion variables, the Bayesian approach obtained the best predictive in men. In women, models using three different methods but not the stepwise Akaike information criterion (AIC) obtained similar AUC values between 0.82 and 0.83. The use of combined facial complexion variables slightly improved the predictive power of hypertension in all four of the models compared with the use of individual variables.

## 1. Introduction

Hypertension has been a persistent worldwide health issue among adults [1,2,3,4,5,6]. The high prevalence of hypertension in various countries and regions had been widely reported, with an estimation of more than a quarter of the adult population in the world having hypertension [7]. In Korea, hypertension is also a crucial public health challenge among both men and women [8,9,10,11,12]. Therefore, various studies have been conducted to determine the predictors of hypertension, including age, gender, body mass index (BMI), anthropometric indices, ethnic groups, cultural groups, physical activities, socioeconomic status, sodium intake, potassium intake, alcohol consumption, and cigarette consumption [13,14,15]. 

Among these, skin color was associated with hypertension. Over the past decades, many studies have been conducted to elucidate the association between hypertension and skin color. Recent studies have shown that blood pressure can be associated with skin color, indicating that individuals with darker skin color are more susceptible to hypertension [16,17]. There have also been studies on the association between skin color and both systolic and diastolic blood pressure [18]. However, prediction of hypertension using skin color indices remains notorious due to the potential confounding factors, including economic status, ethnicity, social classification, age, and gender [16,19,20,21]. Although there are several studies on the association between skin color and hypertension, there are no studies that attempted to predict hypertension using facial complexion as a predictor.

This study aimed to investigate the association between hypertension and facial complexion and determine whether facial complexion are potential predictors for hypertension. We examined the associations between individual complexion indices, combined complexion indices, and hypertension, along with an assessment of their predictive powers. This study was conducted based on actual clinical data and LAB color space was used in the analysis of facial complexion.

## 2. Materials and Methods

### 2.1. Subjects

The data analyzed in this study were obtained from the Korean medicine Data Center (KDC) of the Korea Institute of Oriental Medicine (KIOM). All subjects were recruited from several Oriental medical clinics in Korea from November 2006 to August 2012. Among 3849 subjects recruited, 1099 subjects (376 men and 723 women) were included in this analysis, and the remaining subjects were excluded due to the technical error in the facial variables extraction. All subjects signed an informed consent form, and this study was approved by the KIOM Institutional Review Board (I-0910/02-001).

### 2.2. Definition of Hypertension

For the diagnosis of hypertension, the ‘2018 Korean society of hypertension guideline for the management of hypertension’ was referred to, where hypertension is defined using a threshold of >140/90 mmHg [22]. According to this guideline, a blood pressure (BP) reading of 140/90 mmHg or higher, is classified as hypertensive and BP readings of <120 mmHg and <80 mmHg are classified as normal. 

In this study, the hypertension group included subjects with systolic BP readings of ≥140 mmHg and/or diastolic BP readings of ≥90 mmHg as well as physician-diagnosed hypertension. Physician-diagnosed hypertension defined the subjects who were diagnosed with hypertension at least once by a doctor but did not take any medications or were currently taking antihypertensive medications to control their BP. Those subjects with BP readings of <120 mmHg and <80 mmHg, including those who had fully recovered from hypertension through treatment, were classified in the normal group. 

### 2.3. Extraction of Facial Complexion Variables

#### 2.3.1. Photography

**Figure 1:**
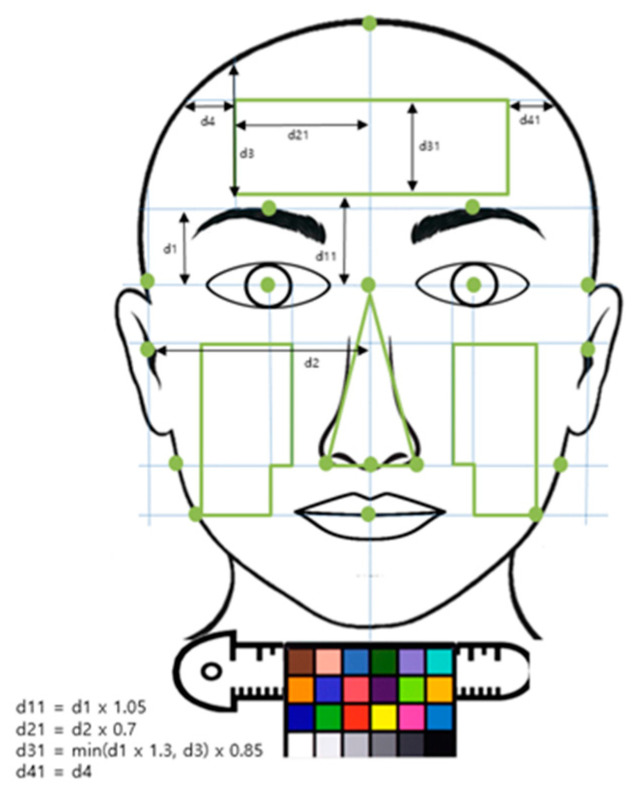
Three regions of facial complexion (forehead, cheek, and nose) [23]. Light green lines and nodes show the grid and facial landmarks used for the construction of facial regions, along with a color chart used for color correction placed below the chin.

A frontal full-face photograph was taken with a neutral expression using a Nikon D700/D5100 digital camera with an 85 mm lens under bilateral illumination at a fixed subject-camera distance of 1.6 m. A color chart used for color correction was attached to a ruler that was placed approximately 1 cm below the chin (Figure 1) [23]. All subjects were requested to present their natural fresh-face without any makeup. Those with makeup are politely asked to remove their makeup and wash their faces before photography. To reduce time period bias, photography of subjects was performed at all seasons throughout the year.

#### 2.3.2. Image Processing

Images were color corrected using a color conversion model that was generated by analyzing the relationship between color information extracted from the color chart of the subject’s image and the reference color information of each cell in the color chart. Three complexion regions (forehead, cheek, and nose) were defined using facial landmarks that were detected automatically using image processing technique (Figure 1). 

#### 2.3.3. Extraction of Facial Complexion Variables

**Figure 2:**
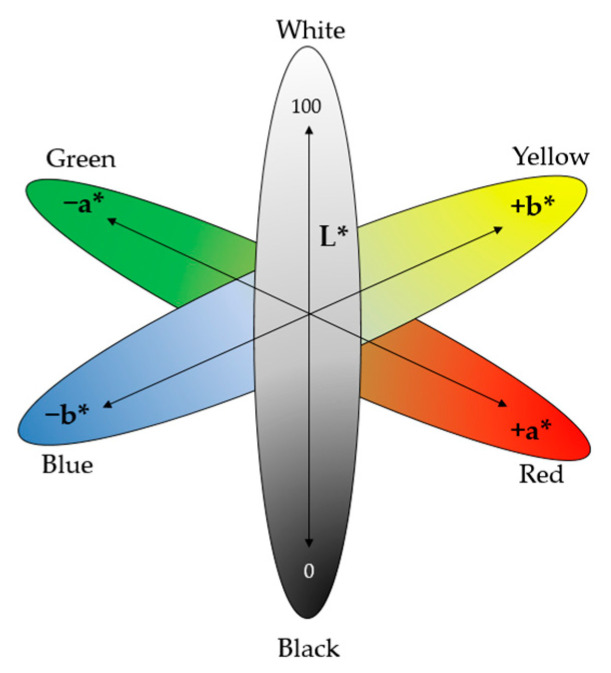
CIELAB color space [24]. L* for lightness, a* and b* for red, green, blue, and yellow of human vision. ’*’ serves as an indication for the Euclidean distance of two color stimuli specified in CIELAB.

Due to the sensitivity on illumination changes, the red (R), green (G), and blue (B) values of each pixel in the complexion regions were extracted and converted into L*, a*, b* values. L*, a*, b* values express color numerically; L* values represent lightness, while a* and b* values represent green-red and blue-yellow color components, respectively (Figure 2). The mean value of L*, a*, and b* in each pixel of the complexion region was calculated and used in the analysis of this study. The complexion variables used in the analysis are described in Table 1. 

### 2.4. Statistical Analysis

#### 2.4.1. Association Between Individual Complexion Variables and Hypertension

All statistical analyses were conducted with R-3.3.2 (Sincere Pumpkin Patch). Before assessing the association between hypertension and facial complexion, two-sample *t*-tests were performed for both genders to compare the differences in general characteristics and complexion variables between the hypertensive group and the normal group. To analyze the association between hypertension and individual complexion variables, binary logistic regression with the individual complexion variable as a predictor was performed with and without adjusting age and BMI after the data were transformed by standardization. The receiver operating characteristic (ROC) curve analysis was used to evaluate the accuracy of the prediction models and to determine which complexion variable was the best indicator and had the best predictive power.

#### 2.4.2. Prediction Models of Hypertension Using Combined Complexion Variables

To assess the association between hypertension and combined complexion variables as well as to compare the predictive powers, Stepwise Akaike information criterion (AIC), as variable subset selection method in logistic regression with adjusted age and BMI, was applied for selecting reliable combined indices and reducing model complexity after data were transformed by standardization, so forth for Least Absolute Shrinkage and Selection Operator (LASSO); the empirical Bayesian LASSO with two-level hierarchical prior, Normal and Exponential distribution (EBLASSO-NE); the empirical Bayesian LASSO with three-level hierarchical prior, Normal, Exponential and Gamma distribution (EBLASSO-NEG) from fully Bayesian approach.

The Stepwise AIC selection was applied using the R function stepAIC, which is a method of choosing a model by AIC in a Stepwise Algorithm. LASSO selection was used as one of the modeling methods, which fits a generalized linear model via a penalized maximum likelihood [24,25]. The LASSO calculation for selecting variables and estimating coefficients in the objective function for the penalized logistic regression is given by Equation (1) [26]:

(1) $$\underset{\beta_{0},\beta_{1}}{\min}-\left[\frac{1}{N}\overset{N}{\underset{i=1}{\sum }}y_{i}\left(\beta_{0}+x_{i}{}^{T}\beta \right)-\log\left(1+e^{\left(\beta_{0}+x_{i}{}^{T}\beta \right)}\right)\right]+\lambda {\left\|\beta \right\|}_{1}$$

where *y_i_* is the status of hypertension, *χ_i_* is the matrix of combined complexion variables, *λ* is a tuning parameter, *β* is the regression coefficient, and *N* is the number of subjects [24,26]. The LASSO penalty selects some of the predictors and discards the others if they are correlated. In this study, the tuning parameter *λ* in the objective function of LASSO was obtained using the R function cv.glmnet, which is the function used to perform cross-validation for tuning selection. The methods of EBLASSO-NE and EBLASSO-NEG are the empirical Bayesian methods for shrinkage of the coefficients and selection of variables for a generalized linear model [27,28]. EBLASSO-NE has a two-level hierarchical prior distribution that is equivalent to the LASSO penalty term, which is in Equation (2) [27]:

(2) $$\beta_{j}\sim N\left(0,\;\sigma_{j}^{2}\right),\;\sigma_{j}^{2}\sim \exp\left(\lambda \right),\;j=1,\cdots ,\;p$$

where *p* is the number of predictors. Estimates by EBLASSO-NE are obtained by maximizing the marginal posterior distribution of *σ*^2^, which is equivalent to maximizing the penalized likelihood function of *σ*^2^, while estimates by LASSO are obtained by maximizing the marginal posterior distribution of *β*, which is equivalent to maximizing the penalized likelihood function of *β* [27,29]. EBLASSO-NEG has a three-level hierarchical prior distribution, which is Equation (3) [27]:

(3) $$\beta_{j}\sim N\left(0,\;\sigma_{j}^{2}\right),\;\sigma_{j}^{2}\sim \exp\left(\lambda \right),\;\lambda \sim gamma\left(a,\;b\right),\;j=1,\cdots ,\;p$$

The estimates calculated by EBLASSO-NEG are obtained by an empirical Bayesian approach, which is similar to the EBLASSO-NE method. The prior distribution in EBLASSO-NE can select variables with a relatively small effect size while the prior in EBLASSO-NEG selects variables with a relatively large effect size [27]. Therefore, the selection by EBLASSO-NEG is recommended when there are many candidate predictors. In this study, the hyperparameters *λ* in EBLASSO-NE and a,b in EBLASSO-NEG were obtained using the R function cv.EBglmnet, which is a function used to perform cross-validation for hyperparameter selection [27,30].

The values of the area under the ROC curve (AUC) are commonly used to evaluate the performance of a prediction model and to compare the performances of competing prediction models in the field of clinical medicine. We calculated the values of AUC and the confidence intervals of AUC for the prediction models by four prediction methods using 5-fold cross-validation, which are as follows: Stepwise AIC, LASSO, EBLASSO-NE, and EBLASSO-NEG. In this study, we obtained the estimates and confidence intervals of AUC using the R function ci.cvAUC, which calculates the influence curve-based confidence intervals for cross-validated AUC estimates [31,32].

## 3. Results

### 3.1. General Characteristics and Complexion Variables of the Subjects

The general characteristics and complexion variables of the subjects are summarized in Table 2. Among the 1099 subjects with an age range of 15–90 years included in this analysis, there were 394 subjects in the hypertensive group and 705 subjects in the normal group. In the hypertensive group, 53.76% of men and 64.42% of women were currently treated with antihypertensive medication. Significant differences between the hypertensive group and the normal group were observed in most of the general characteristics of both men and women. 

The Total_L*, Fh_L*, Ch_L* and Ns_L* were significantly lower in the hypertensive group than in the normal group in both genders (*p* = 0.003, 0.008, 0.011 and 0.014, respectively, in men; *p* < 0.001, <0.034, <0.001 and <0.001, respectively, in women). As L* values are defined by lightness, the hypertensive group had darker and duller facial complexions than the normal group for the total face area, forehead, cheek, and neck region. 

For the a* value, which extends from green to red, the Total_a*, Fh_a*, Ch_a* and Ns_a* were significantly higher in the hypertensive group than in the normal group in both genders (*p* = 0.001, <0.001, <0.006 and <0.003, respectively in men; *p* < 0.001, <0.001, <0.001 and <0.001, respectively, in women). These results indicate that the facial complexion of subjects in the hypertensive group tends to be darker and redder than that in the normal group. 

The Total_b*, Fh_b*, Ch_b*, and Ns_b* were significantly higher in the hypertensive group than in the normal group in women, showing that hypertensive women had a more yellow facial complexion than non-hypertensive women. There were no significant differences found in the b* value for men.

### 3.2. Association Between Individual Complexion Variables and Hypertension

The results for the association between individual complexion variables and hypertension with their predictive powers, with and without adjusted age and BMI, were shown in Table 3 and Table 4. 

For men, BMI exhibited the strongest association with hypertension (*p* < 0.001, OR (95% CI) = 2.202 (1.72–2.864), AUC (95% CI) = 0.698 (0.645–0.75)), followed by age (*p* < 0.001, OR = 1.174 (1.406–2.176), AUC = 0.649 (0.595–0.704)) in crude analysis. These results suggest that age and BMI were strong predictors of hypertension. In terms of individual complexion variables, Fh_a* (*p* < 0.001, OR = 1.663 (1.304–2.142), AUC = 0.615 (0.559–0.672)), Total_a* (*p* = 0.001, OR = 1.491 (1.187–1.889), AUC = 0.595 (0.538–0.652)) and Ns_a* (*p* = 0.003, OR = 1.479 (1.142–1.929), AUC = 0.595 (0.538–0.652)), were identified as strong indicators in crude analysis. In the crude analysis, all of the L* and a* variables exhibited associations with hypertension except for b* variables, which indicates that men with a dark and red facial complexion might have a higher probability of having hypertension. However, all associations between individual complexion indices and hypertension disappeared after adjusting for age and BMI.

Similar to men, age and BMI were strong predictors for women (age, *p* < 0.001, OR = 3.832 (3.062–4.87), AUC = 0.804 (0.769–0.838); BMI, *p* < 0.001, OR = 2.127 (1.786–2.553), AUC = 0.706 (0.665–0.747)) in crude analysis. All the individual complexion variables in crude analysis were associated with hypertension. The complexion variables with the highest associations were the variables a* for green–red color, followed by L* for lightness and b* for blue-yellow in all facial regions. These findings indicate that women with red yellow and dark complexions might have a higher probability of having hypertension. After adjusting for age and BMI, Total_L* (*p* = 0.007, OR = 0.746 (0.601–0.921), AUC = 0.83 (0.798–0.862)), and Ns_L* (*p* = 0.004, OR = 0.752 (0.617–0.913), AUC = 0.83 (0.799–0.862)) became the strongest predictors but the associations between the complexion variable b* and hypertension disappeared. 

Age and BMI were strong predictors in both genders. In crude analysis, the a* variables among all complexion variables had the highest associations with hypertension in all facial regions and were significant predictors in both men and women. After adjusting for age and BMI, the complexion variables L* and a* in all facial regions remained highly associated with hypertension and had strong predictive powers in the women, whereas all variable associations disappeared in the men. 

### 3.3. Comparison of the Predictive Powers for Hypertension Using Combined Complexion Variables

The results of the predicting models using the combined complexion variables as predictors for hypertension and their predictive powers based on four variable subset selection methods in logistic regression (Stepwise AIC, LASSO, EBLASSO-NE, and EBLASSO-NEG) are presented in Table 5. We also performed 5-fold cross-validation to obtain estimates and confidence intervals for the AUC. 

**Figure 3:**
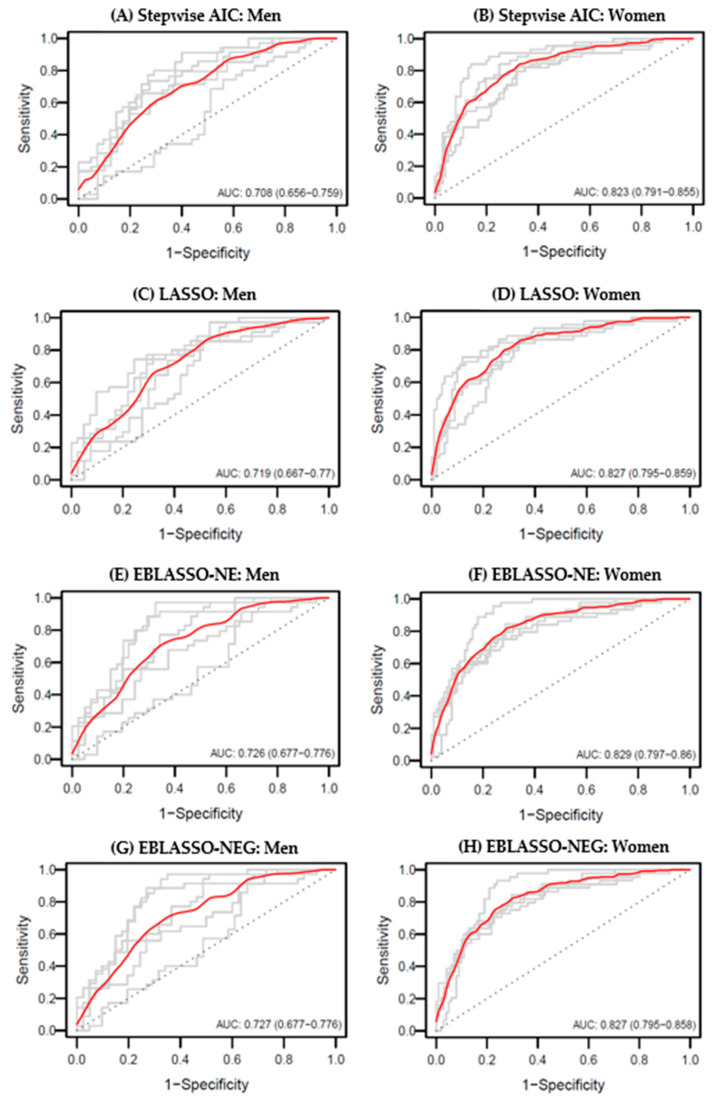
Roc curves for (**A**) Stepwise AIC model of men, (**B**) Stepwise AIC model of women, (**C**) LASSO model of men, (**D**) LASSO model of women, (**E**) EBLASSO-NE model of men, (**F**) EBLASSO-NE model of women, (**G**) EBLASSO-NEG of men, and (**H**) EBLASSO-NEG of women using 5-fold cross-validation. Grey lines show individual ROC curves for 5-fold cross-validation, and the red line shows the average of individual ROC curves. LASSO, least absolute shrinkage and selection operator; Stepwise AIC, Stepwise Akaike information criterion; EBLASSO-NE, the empirical Bayesian LASSO with two-level hierarchical prior, Normal and Exponential distribution; EBLASSO-NEG, the empirical Bayesian LASSO with three-level hierarchical prior, Normal, Exponential and Gamma distribution; AUC, area under the receiver operating characteristic curve using 5-fold cross-validation; ROC, receiver operating characteristic.

For men, the AUC values of the EBLASSO-NE and EBLASSO-NEG predicting models (EBLASSO-NE, AUC = 0.726 (0.677–0.776); EBLASSO-NEG, AUC = 0.727 (0.677–0.776)) were higher than the other models. For women, all four predicting models had similar AUC values between 0.82 and 0.83, but the AUC value of the Stepwise AIC predicting model was slightly lower (AUC = 0.823 (0.791–0.855)). On the other hand, the AUC values of the models using combined variables were higher than the AUCs of the models using individual variables without adjustment and similar to the AUCs of the models using individual variables with adjustment in both genders. These results demonstrate that the predictive power can be improved slightly by using combined complexion variables as predictors for hypertension. The ROC curves displaying the variable subset selection methods are shown in Figure 3.

The EBLASSO-NE predicting model selected five variables, BMI, Age, Fh_L*, Fh_a*, and Ch_b* among the combined complexion variables in men, indicating that this model includes these variables as predictors for hypertension. The LASSO and EBLASSO_NE predicting model in women selected the same complexion variables, BMI, Age, Total_L*, Fh_a*, Ns_L*, and Ns_a*, as predictors. Both EBLASSO-NEG models in men and women selected the least predictors as compared to other predicting models. Excluding age and BMI, the four predicting models in men only included a few complexion variables, similar to the results in the adjusted analysis in Table 3. For women, all four predicting models selected L* and a* variables, similar to the results using individual variables shown in Table 4.

## 4. Discussion

Our study obtained several interesting results after statistically analyzing the associations between facial complexion variables and hypertension. In this study, facial complexion, age, and BMI were used as variables to predict hypertension. The CIELab is used for the variable of facial complexion as it is a color space modeled on the visual pattern of humans and designed to be perceptually uniform in human perceptual work [33]. 

Our results reveal that age and BMI are the best predictors for hypertension in both men and women. The probability of having hypertension is shown to increase by approximately 1.741 times for men and approximately 3.832 times for women along with aging. These findings are consistent with the results of previous studies, where age and BMI are the risk factors of hypertension [34,35,36,37]. For facial complexion variables, our findings reveal that the L* and a* variables in men and all L*, a*, and b* variables in women showed associations with hypertension, with a* variables as the strongest predictors in both genders. After adjusting for age and BMI, only L* and a* variable in women remained associated with hypertension whereas no variable in men showed associations. 

Previous studies have shown that CIELab a* values can be influenced by blood in human skin, and skin redness could also be increased by increased a* values [38,39]. In our study, 53.76% of hypertensive men and 67.42% of hypertensive women were under antihypertensive medication. Among the five classes of antihypertensive medication, calcium channel blockers are one of the recommended first-line treatments in Korea [40]. According to the study by Russell, flushing and vasodilation are the majority clinical side effects of calcium channel blockers [41]. A previous study has also reported that calcium channel blockers can induce cutaneous reactions [42]. One Taiwanese study has also revealed hypertension is associated with rosacea, where rosacea might be triggered by high BP [43]. These studies might be able to corroborate and explain our findings in which a* variables are identified to be strong predictors in all facial regions in the crude analysis for both genders in this study.

Besides, four models are built using different variable selection methods, Stepwise AIC, LASSO, EBLASSO-NE and EBLASSO-NEG, to examine the association between hypertension and combined complexion variables and their predictive powers. The predicting power of all four predicting models is higher in women than in men, with the combined complexion variable having higher predicting power than individual complexion variables. In our study, the LASSO and EBLASSO_NE predicting model in women selected similar complexion variables as hypertension predictors. This might be due to the two predicting models having the same penalty term. In the meantime, the EBLASSO_NEG predicting model selected very few variables as predictors in both men and women because this model discards variables with relatively small effect size and selects variables with a relatively large effect size. Looking at our results, combined facial complexion variables have great potential as a predictor for hypertension in women. 

To the best extent of our knowledge, this is the first study to propose facial complexion as a predictor for hypertension. Several studies in the past have examined the relationship between skin color and BP using data derived from skin color. Boyle described a linear increase in BP with increasing skin darkness among black men and women in a cohort study [44]. After adjusting for confounding factors, Keil et al. suggested that there is no relationship between skin color and BP in the follow-up studies of this cohort [45,46]. In the study of Ernst et al., there was a positive association between SBP and skin darkness in black populations [47]. However, Klag et al. mentioned that such a linear relationship only occurs among black men and women of low socioeconomic status [48]. Hence, the association between skin darkness and BP remains controversial. Our study agrees with the premise that there is an association between skin darkness and BP, as our results show that individuals with darker and duller facial complexions have a higher probability of having hypertension by expressing lower odds ratios of L* values than 1 in women. Despite this, our study might have limited contribution to the comprehension of association between skin color and hypertension, as we focused our study on facial region only and did not include other factors such as cumulative sun exposure, skin pigmentation, nationality, education level, and ethnicity. 

Although our statistical findings show that the facial complexion variable differences between hypertensive and normal groups are rather small, the effect sizes for several variables in this study with account of inter-class variation are computed to be medium effect sizes (d ≈ 0.5). The effect sizes for the remaining variables are relatively small. In studies that inquire about new areas of research, effect sizes are probable to be small (d = 0.2) due to the lack of refinement in experimental and measurement controls [49]. Therefore, the effect sizes for our results are generally conceived to be large enough to be apparent and the performance of our predictive models are also fairly good. 

There are several limitations to this study. First, only data of 1099 subjects from 3849 subjects were included in this study. Many data were excluded due to excessive facial hairs in men and application of makeup in women, in which subjects that refused to shave or remove their makeup were marked and excluded in the analysis, as their photographs could cause technical errors and alter the measurements in their facial variables extraction. This also leads to the unbalanced sample size of men and women in this study, which might introduce certain biases in our findings. Second, data from this study was collected by different technicians because the subjects were recruited from multiple sites across the country. Although all technicians were well-trained and followed a standard procedure, inconsistency may nonetheless exist in the data collection due to individual differences. Therefore, our future studies should have a balanced sample size, and the consistency in data collection should be increased. Third, there might be several confounding factors that we did not consider in this study. To increase the validity and generalization of our results, another known determinant of hypertension such as cumulative sun exposure should be taken into account and this study could be extended by conducting the study on non-sun exposed skin. Future studies should also include time of the year for data sampling as a factor as facial complexion may change with the seasons. 

## 5. Conclusions

By using the CIELab color system, our study revealed the role of facial complexion in predicting hypertension. We found that facial complexion seems to be a better predictor of hypertension in women than in men, with L* and a* values as the main variables. As this is an exploratory study, this study may serve as a foundation and further studies are needed to validate the potential of facial complexion as a predictor for hypertension.

## Figures and Tables

**Table 1:** Description of the complexion variables.

Variable Name	Description
Total_L*	L* value in the total face for lightness
Total_a*	a* value in the total face for green-red color components
Total_b*	b* value in the total face for blue-yellow color components
Fh_L*	L* value in the forehead for lightness
Fh_a*	a* value in the forehead for green-red color components
Fh_b*	b* value in the forehead for blue-yellow color components
Ch_L*	L* value in the cheek for lightness
Ch_a*	a* value in the cheek for green-red color components
Ch_b*	b* value in the cheek for blue-yellow color components
Ns_L*	L* value in the nose for lightness
Ns_a*	a* value in nose for green-red color components
Ns_b*	b* value in nose for blue-yellow color components

The variables were calculated in L*a*b* color space. ’*’ serves as an indication for the Euclidean distance of two color stimuli specified in CIELAB.

**Table 2:** General characteristics and complexion variables of the subjects.

Variable	Men			Women		
	Normal	Hypertensive	*p*-Value	Normal	Hypertensive	*p*-Value
Subjects	203	173	−	502	221	−
Age	43.36 ± 16.76	51.68 ± 13.05	<0.001	43.85 ± 13.8	59.7 ± 12.64	<0.001
Height [cm]	170.54 ± 6.42	170.62 ± 6.35	0.908	158.86 ± 5.67	155.54 ± 5.75	<0.001
Weight [kg]	67.5 ± 9.83	73.44 ± 10.31	<0.001	56.57 ± 8	59.94 ± 9.1	<0.001
BMI [kg/m^2^]	23.18 ± 2.86	25.19 ± 2.89	<0.001	22.42 ± 2.97	24.75 ± 3.27	<0.001
SBP [mmHg]	115.03 ± 11.3	132.94 ± 15.53	<0.001	110.45 ± 12.01	130.77 ± 16.39	<0.001
DBP [mmHg]	73.02 ± 8.94	86.47 ± 11.13	<0.001	70.98 ± 8.65	83.78 ± 11.44	<0.001
Receiving treatment (%)	−	53.76	−	−	67.42	−
Pulse rate [bpm]	72.01 ± 9.96	70.97 ± 9.39	0.299	72.23 ± 9.62	75.06 ± 11.84	0.002
Temperature [°C]	36.28 ± 0.35	36.28 ± 0.35	0.921	36.38 ± 0.34	36.36 ± 0.4	0.500
Total_L*	60.98 ± 4.45	59.66 ± 4.13	0.003	65.73 ± 3.97	64.31 ± 4.81	<0.001
Total_a*	13.9 ± 2.26	14.73 ± 2.32	0.001	11.57 ± 2.12	12.61 ± 2.25	<0.001
Total_b*	21.24 ± 2.35	21.02 ± 2.35	0.369	20.87 ± 2.72	21.52 ± 2.85	0.004
Fh_L*	66.38 ± 5.31	65.01 ± 4.66	0.008	70.39 ± 4.94	69.48 ± 5.48	0.034
Fh_a*	12.78 ± 2.45	13.82 ± 2.35	<0.001	9.94 ± 2.3	11.09 ± 2.43	<0.001
Fh_b*	22.05 ± 2.7	22.04 ± 2.66	0.961	21.33 ± 3.07	22.12 ± 3.22	0.002
Ch_L*	57.32 ± 4.62	56.14 ± 4.26	0.011	62.46 ± 3.81	61 ± 4.79	<0.001
Ch_a*	14.46 ± 2.32	15.16 ± 2.55	0.006	12.59 ± 2.17	13.49 ± 2.31	<0.001
Ch_b*	20.77 ± 2.39	20.42 ± 2.46	0.169	20.64 ± 2.74	21.21 ± 2.87	0.012
Ns_L*	66 ± 5.15	64.69 ± 5.08	0.014	70.47 ± 5.45	68.66 ± 5.87	<0.001
Ns_a*	15.68 ± 2.56	16.48 ± 2.65	0.003	11.68 ± 2.53	12.8 ± 2.82	<0.001
Ns_b*	20.63 ± 2.43	20.51 ± 2.59	0.661	19.92 ± 3.03	20.71 ± 3.07	0.002

BMI, Body mass index; SBP, systolic blood pressure; DBP, diastolic blood pressure; ’− ‘, not applicable; ’*’, indication for the Euclidean distance of two color stimuli specified in CIELAB. Receiving treatment (%) is defined as the percentage of subjects currently under antihypertensive medication. Data are represented in the mean ± SD (standard deviation). *p*-values were obtained from independent two-sample *t*-tests between the normal group and the hypertensive group.

**Table 3:** The associations between individual complexion variables and hypertension in men with their predictive powers.

Variable	Crude			Adjusted		
	*p*-Value	OR (95% CI)	AUC (95% CI)	*p*-Value	OR (95% CI)	AUC (95% CI)
Age	<0.001	1.741 (1.406–2.176)	0.649 (0.595–0.704)	−	−	−
BMI	<0.001	2.202 (1.72–2.864)	0.698 (0.645–0.75)	−	−	−
SBP	<0.001	6.032 (4.162–9.127)	0.821 (0.774–0.868)	<0.001	5.526 (3.752–8.509)	0.855 (0.817–0.893)
DBP	<0.001	5.07 (3.634–7.351)	0.836 (0.789–0.883)	<0.001	6.115 (4.155–9.424)	0.880 (0.845–0.915)
Total_L*	0.004	0.703 (0.551–0.889)	0.588 (0.531–0.646)	0.434	0.899 (0.688–1.172)	0.733 (0.683–0.782)
Total_a*	0.001	1.491 (1.187–1.889)	0.595 (0.538–0.652)	0.741	1.046 (0.8–1.368)	0.734 (0.684–0.783)
Total_b*	0.368	0.9 (0.715–1.131)	0.452 (0.394–0.51)	0.189	0.846 (0.656–1.085)	0.726 (0.676–0.775)
Fh_L*	0.009	0.738 (0.585–0.925)	0.579 (0.521–0.636)	0.221	0.855 (0.665–1.097)	0.733 (0.683–0.782)
Fh_a*	<0.001	1.663 (1.304–2.142)	0.615 (0.559–0.672)	0.343	1.148 (0.864–1.529)	0.736 (0.687–0.785)
Fh_b*	0.961	0.994 (0.792–1.248)	0.435 (0.377–0.493)	0.487	0.916 (0.713–1.174)	0.723 (0.673–0.773)
Ch_L*	0.012	0.744 (0.588–0.935)	0.578 (0.52–0.636)	0.762	0.96 (0.737–1.248)	0.731 (0.682–0.78)
Ch_a*	0.007	1.345 (1.089–1.672)	0.578 (0.52–0.636)	0.984	1.002 (0.784–1.279)	0.732 (0.683–0.782)
Ch_b*	0.168	0.853 (0.678–1.068)	0.55 (0.491–0.608)	0.100	0.813 (0.634–1.039)	0.727 (0.677–0.777)
Ns_L*	0.015	0.744 (0.583–0.942)	0.583 (0.525–0.641)	0.282	0.866 (0.665–1.124)	0.734 (0.685–0.784)
Ns_a*	0.003	1.479 (1.142–1.929)	0.595 (0.538–0.652)	0.932	1.013 (0.75–1.366)	0.730 (0.681–0.78)
Ns_b*	0.658	0.948 (0.749–1.199)	0.466 (0.408–0.524)	0.386	0.891 (0.686–1.155)	0.724 (0.675–0.774)

OR, Odds Ratio; AUC, area under the receiver operating characteristic curve; CI, confidence interval; ’− ‘, not applicable; ’*’, indication for the Euclidean distance of two color stimuli specified in CIELAB. The statistical analysis of the data was performed using logistic regression with individual complexion variables as predictors after the data were transformed by standardization. The ORs and *p*-values in the adjusted parts of the table were adjusted by age and BMI. The values of AUC and confidence intervals of AUC using 5-fold cross-validation were calculated using the influence curve [31].

**Table 4:** The associations between individual complexion variables and hypertension in women with their predictive powers.

Variable	Crude			Adjusted		
	*p*-Value	OR (95% CI)	AUC (95% CI)	*p*-Value	OR (95% CI)	AUC (95% CI)
Age	<0.001	3.832 (3.062–4.87)	0.804 (0.769–0.838)	−	−	−
BMI	<0.001	2.127 (1.786–2.553)	0.706 (0.665–0.747)	−	−	−
SBP	<0.001	6.417 (4.833–8.735)	0.84 (0.805–0.874)	<0.001	4.824 (3.559–6.71)	0.895 (0.871–0.919)
DBP	<0.001	5.584 (4.228–7.555)	0.815 (0.776–0.853)	<0.001	5.257 (3.848–7.405)	0.906 (0.883–0.928)
Total_L*	<0.001	0.677 (0.558–0.817)	0.58 (0.533–0.627)	0.007	0.746 (0.601–0.921)	0.830 (0.798–0.862)
Total_a*	<0.001	1.727 (1.434–2.09)	0.639 (0.595–0.683)	0.018	1.302 (1.047–1.622)	0.828 (0.797–0.86)
Total_b*	0.004	1.252 (1.076–1.46)	0.571 (0.525–0.616)	0.560	1.056 (0.88–1.267)	0.824 (0.792–0.856)
Fh_L*	0.028	0.823 (0.691–0.977)	0.539 (0.492–0.585)	0.020	0.789 (0.645–0.962)	0.829 (0.797–0.861)
Fh_a*	<0.001	1.785 (1.472–2.176)	0.646 (0.602–0.689)	0.010	1.342 (1.073–1.685)	0.828 (0.796–0.86)
Fh_b*	0.002	1.273 (1.093–1.485)	0.57 (0.525–0.616)	0.668	1.041 (0.867–1.252)	0.824 (0.791–0.856)
Ch_L*	<0.001	0.65 (0.531–0.791)	0.593 (0.546–0.639)	0.017	0.763 (0.609–0.95)	0.829 (0.797–0.861)
Ch_a*	<0.001	1.557 (1.304–1.867)	0.616 (0.572–0.66)	0.060	1.223 (0.992–1.51)	0.827 (0.795–0.859)
Ch_b*	0.011	1.221 (1.048–1.425)	0.562 (0.516–0.607)	0.473	1.069 (0.89–1.285)	0.824 (0.792–0.856)
Ns_L*	<0.001	0.707 (0.593–0.838)	0.588 (0.542–0.633)	0.004	0.752 (0.617–0.913)	0.83 (0.799–0.862)
Ns_a*	<0.001	1.681 (1.379–2.057)	0.617 (0.572–0.662)	0.007	1.381 (1.096–1.748)	0.829 (0.798–0.861)
Ns_b*	0.002	1.274 (1.097–1.487)	0.57 (0.524–0.615)	0.606	1.049 (0.877–1.258)	0.824 (0.792–0.856)

OR, Odds Ratio; AUC, area under the receiver operating characteristic curve; CI, confidence interval; ’− ‘, not applicable; ’*’, indication for the Euclidean distance of two color stimuli specified in CIELAB. The statistical analysis of the data was performed using logistic regression with individual complexion variables as predictors after the data were transformed by standardization. The ORs and *p*-values in the adjusted parts of the table were adjusted by age and BMI. The values of AUC and confidence intervals of AUC using 5-fold cross-validation were calculated using the influence curve [31].

**Table 5:** The variables selected by Stepwise AIC, LASSO, EBLASSO-NE, and EBLASSO-NEG methods in logistic regression and the powers of the models.

Variable Subset Selection Method	Number of Selected Variables	Selected Variables	AUC (95% CI)
*Men*			
Stepwise AIC	3	BMI, Age, Ch_b*	0.708 (0.656–0.759)
LASSO	3	BMI, Age, Fh_a*	0.719 (0.667–0.77)
EBLASSO-NE	5	BMI, Age, Fh_L*, Fh_a*, Ch_b*	0.726 (0.677–0.776)
EBLASSO-NEG	2	BMI, Age	0.727 (0.677–0.776)
*Women*			
Stepwise AIC	6	BMI, Age, Total_L*, Fh_L*, Fh_a*, Ch_L*	0.823 (0.791–0.855)
LASSO	6	BMI, Age, Total_L*, Fh_a*, Ns_L*, Ns_a*	0.827 (0.795–0.859)
EBLASSO-NE	6	BMI, Age, Total_L*, Fh_a*, Ns_L*, Ns_a*	0.829 (0.797–0.86)
EBLASSO-NEG	4	BMI, Age, Fh_a*, Ns_L*	0.827 (0.795–0.858)

LASSO, least absolute shrinkage and selection operator; Stepwise AIC, Stepwise Akaike information criterion; EBLASSO-NE, the empirical Bayesian LASSO with two-level hierarchical prior, Normal and Exponential distribution; EBLASSO-NEG, the empirical Bayesian LASSO with three-level hierarchical prior, Normal, Exponential and Gamma distribution; AUC, area under the receiver operating characteristic curve using 5-fold cross-validation; CI, confidence interval; ’*’, indication for the Euclidean distance of two color stimuli specified in CIELAB. The statistical analysis of the data was performed using logistic regression with combined complexion variables as predictors after the data were transformed by standardization. Stepwise AIC, LASSO, EBLASSO-NE, and EBLASSO-NEG were used as variable selection methods. The values of AUC and confidence intervals of AUC using 5-fold cross-validation were calculated using the influence curve [31].

## Data Availability

The data used to support the findings of this study are available from the corresponding author upon request.
